# Nephron index rather than serum FGF 23 predicts endothelial dysfunction in early but not advanced chronic kidney disease patients

**DOI:** 10.1007/s11255-023-03589-y

**Published:** 2023-04-12

**Authors:** Nora Khreba, Doaa Khedr, Azza Abdel-Baky, Ghada El Kannishy, Emad Samaan

**Affiliations:** 1 Mansoura Nephrology and Dialysis Unit, Mansoura Faculty of Medicine, Internal Medicine Depament, Mansoura University, El Gomhoria St., Mansoura, 35516 Egypt; 2 Clinical Pathology Department, Mansoura Faculty of Medicine, Mansoura, Egypt; 3 Diagnostic and Interventional Radiology Department, Mansoura Faculty of Medicine, Mansoura, Egypt

**Keywords:** Nephron index, FGF 23, Urinary phosphorus, Stage 3 CKD, Endothelial dysfunction, FMD

## Abstract

**Background:**

Endothelial dysfunction is the primary step for the development of CKD-related cardiovascular disease. Early prediction and management can influence patient survival. Serum testing of FGF 23 hormone and urinary phosphate excretion were studied as predictors of all-cause cardiovascular morbidity in CKD patients; however, their relation to endothelial dysfunction is controversial. A combination of both in one index is hypothesized to increase their sensitivity in detecting endothelial dysfunction, especially in the early stages of CKD before the dominance of hyperphosphatemia, the original risk.

**Methods:**

A cross-sectional comparative analysis between thirty CKD stage 3 patients and sixty stage 4–5 CKD patients was conducted. All patients were tested for markers of mineral bone disorders including serum FGF 23 and 24-h urinary phosphate excretion. A combination of both in one index (nephron index) is calculated and hypothesized to correlate with nephron number. Endothelial dysfunction was assessed by measuring the post-occlusion brachial flow-mediated dilatation (FMD).

**Results:**

In univariate and multivariate regression analyses, the nephron index was the only predictor of endothelial dysfunction in individuals with stage 3 CKD (*r* = 0.74, *P* 0.01). This was not applied to stage 4–5 CKD patients where serum phosphorus (*r* = − 0.53, *P* 0.001), intact PTH (*r* = − 0.53, *P* 0.001), uric acid (*r* = − 0.5, *P* 0.001), and measured GFR (*r* = 0.59, *P* 0.001) were the highest correlates to FMD; the Nephron index had the weakest correlation (*r* = 0.28, *P* = 0.02) and is not predictive of endothelial dysfunction.

**Conclusion:**

Nephron index calculation showed better correlation with endothelial dysfunction than using any of its determinants alone in early stages of CKD when FGF 23 levels are just beginning to rise. In advanced CKD patients, hyperphosphatemia, hyperparathyroidism, hyperuricemia, and measured GFR are more reliable than nephron index.

## Introduction

The vascular endothelium regulates multiple vital processes, including vascular tone and permeability, inflammatory responses, thrombosis, and angiogenesis. Endothelial dysfunction (ED) is a major pathogenetic inducer of atherosclerosis and cardiovascular disease. Chronic kidney disease (CKD) is characterized by reduced nitric oxide bioavailability, which is almost present in all patients approaching end-stage renal disease (ESRD). Numerous factors can affect the expression of endothelial NO synthase and contribute to the low NO bioavailability in CKD [1]. Among them, is the increased serum fibroblast growth factor 23 (FGF23), which gained great research attention in the last decade as an early risk factor and marker for cardiovascular complications in CKD patients [2]. FGF23 is a bone-derived phosphaturic through its action at the proximal tubules and thus, it is correlated with phosphate excretion per nephron [3]. FGF23 rises during CKD progression as early as stage 2, a long time before the increase in serum phosphate levels, which emerge at stage 4 or later. The gradual rise in FGF23 is thought to be a result of compensation to the declining number of functioning nephrons and to the relative resistance to its action due to klotho deficiency. This must be balanced off by an increase in phosphate excretion per nephron to maintain the phosphate homeostasis [4]. Many observational studies investigated the association between FGF23 and various cardiovascular adverse events in CKD [5] like left ventricular hypertrophy [6], vascular calcification [7], and increased risk of ischemic heart and brain insults [8]. The correlation between FGF23 concentration and markers of atherosclerosis and ED in different CKD, HD, and transplantation patients were studied before in a few reports with controversial results. Most of these reports targeted atherosclerosis more than ED [9, 10]. The ratio of urinary phosphate excretion (mg/day) to serum FGF23 was considered as an index reflecting the functional nephron number, which was defined as the nephron index [11]. The nephron index was reported before to be associated with atherosclerosis in early-stage CKD [12] and diabetic CKD [13]. Since it is anticipated that it will begin to decline in the early stages of CKD, we hypothesize that the nephron index can be a more sensitive predictor of the early ED than serum FGF 23 testing alone. We conducted this cross-sectional study to assess the relationship between the nephron index and ED as measured by the brachial artery flow-mediated dilatation (FMD) in stage 3 CKD in comparison with advanced stages. Percentage of post-occlusion FMD represents a functional assessment of ED [1].

## Materials and methods

This cross-sectional study was conducted on 90 CKD adult patients, who were recruited from Mansoura University Hospital (Internal medicine department) and attended Nephrology Clinic, Mansoura University, over a period of 2 years from 2019 to 2021.

### Patient selection

The patients were selected based on their measured creatinine clearance by 24-h urine collection. CKD diagnosis was established according to KDIGO 2012 guidelines [14] which defined CKD as an abnormality of kidney structure or function, present for > 3 months, with health implications, and requires one of two criteria documented or inferred for > 3 months: either GFR < 60 ml/min/1.73 m^2^ or markers of kidney damage. The study population was grouped according to measured creatinine clearance to Stage 3 CKD (30 patients) with creatinine clearance between 30 and 60 ml/min/1.73 m^2^, and stage 4–5 CKD (60 patients) with creatinine clearance below 30 ml/min/1.73 m^2^. We included CKD patients aged ≥ 18 and $\le$ 60 years who agreed to participate in the study, share their clinical and laboratory data and assign written consent. While patients who had acute renal insult on top of CKD, active malignancy, active infection and/or active inflammatory processes, decompensated heart failure, recent acute coronary event, and those on hemodialysis were excluded.

### Sample size calculation

Calculation relied upon a previous cross-sectional report by Recio-Mayoral A et al. [15]. Using the SD of FMD (2.26), the following equation is used to calculate the sample size:

$$n = Z_{{{\raise0.7ex\hbox{$\alpha $} \!\mathord{\left/ {\vphantom {\alpha 2}}\right.\kern-0pt} \!\lower0.7ex\hbox{$2$}}}}^{2} \left( {\frac{\sigma }{d}} \right)^{2} .$$

The current study sample size guaranteed a 99% confidence estimate of mean FMD with a 0.6% margin of error.

### Data collection

All patients were subjected to history taking, physical examination, and laboratory evaluation including Serum calcium, serum phosphorus, intact parathyroid hormone (iPTH), and total urine collected for 24 h was sent to the laboratory for estimating creatinine clearance and daily phosphate excretion. Serum human fibroblast growth factor-23 (FGF23) was assayed by enzyme-linked immunosorbent assay (ELISA) supplied by SinoGeneClon Biotech Co. The nephron index represents nephron number and is suggested to be correlated positively with the ratio of urinary phosphate excretion (mg/day) to serum FGF23 [11].

$$\mathrm{Phosphate excretion per nephron}=\frac{urinary phosphate excretion (mg/day)}{nephron number}\propto FGF23.$$

$$\therefore \mathrm{Nephron number}\propto \frac{urinary phosphate excretion \left(\frac{mg}{day}\right)}{FGF23}=Nephron index.$$

#### Functional assessment of endothelial dysfunction

**Fig. 1:**
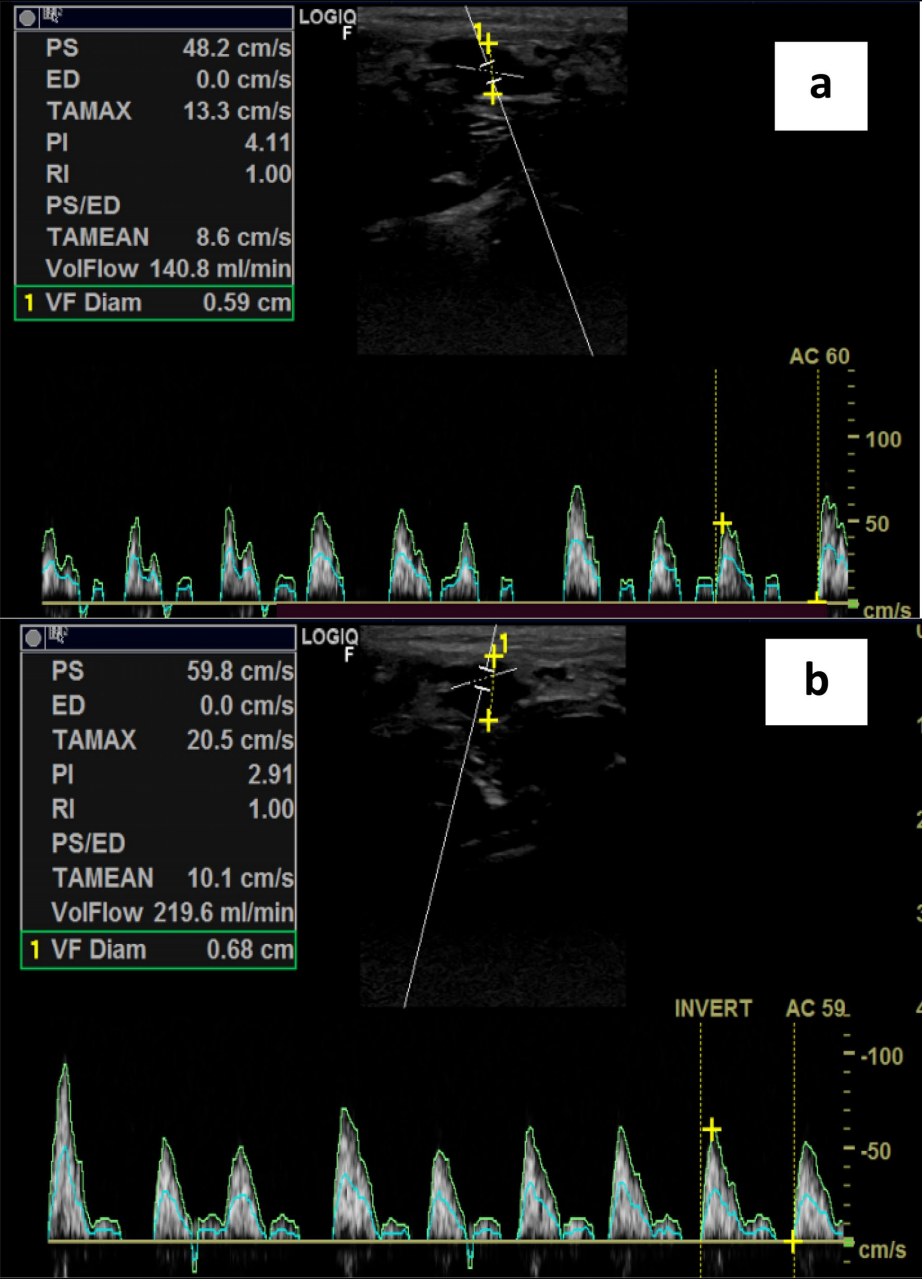
Example of percentage of flow mediated dilatation calculation. **a** B mode pre-hyperemic sonogram of the arm taken 5 cm proximal to the cubital fossa showing the lumen-intima interface diameter measured as predeflation diameter. **b** B mode post-hyperemic sonogram of the arm taken 5 cm proximal to the cubital fossa showing the lumen–intima interface diameter measured as post-deflation diameter

Evaluation of endothelial dysfunction through brachial artery flow-mediated dilatation (FMD) was performed according to the American College of Cardiology guidelines [16]. The brachial artery was scanned 5–15 cm above the antecubital fossa. The resting diameter was measured, and then a blood pressure cuff was inflated around the arm to at least 50 mmHg above systolic blood pressure for 4.5 min. Measurement of maximum diameter was taken 45–60 s after cuff release. FMD was calculated according to [brachial artery diameter post-deflation − baseline brachial artery diameter)/baseline brachial artery diameter] × 100 (Fig. 1).

### Statistical analysis

Data were fed to the computer and analyzed using IBM SPSS Corp. Released in 2013. IBM SPSS Statistics for Windows, Version 22.0. Qualitative data were described using numbers and percentages. Quantitative data were described using median (minimum and maximum) for non-parametric data and mean ± standard deviation for parametric data after testing normality using the Kolmogorov–Smirnov test. For qualitative data Chi-Square test was done for comparing 2 groups, the Student *t* test was used to compare parametric data of independent variables of the 2 groups, and Mann–Whitney *U* test was used to compare non-parametric data of independent variables of the 2 groups. Spearman’s rank-order correlation is used to determine the strength and direction of a linear relationship between two non-normally distributed continuous variables and /or ordinal variables. Multivariable linear regression analysis was done to determine the predictors of endothelial dysfunction in CKD patients. A *P* value less than 0.05 was considered statistically significant.

## Results

Both study groups had comparable age, gender, and comorbidities distribution except for hypertension (HTN) prevalence which is much higher in CKD patients with creatinine clearance below 30 ml/min/1.73 m^2^. Stage 3 CKD patients had significantly higher hemoglobin, serum calcium, and nephron index values. They also have significantly lower phosphate, serum FGF 23, PTH, and uric acid levels. Median value of FMD percentage was much lower in CKD patients with creatinine clearance below 30 (Table 1).

**Table 1:** Comparison of demographic, clinical, laboratory, and radiological criteria between the study groups

	CKD stages		*P*
	3 (*N* = 30)	4–5 (*N* = 60)	
Demographic and clinical data			
Age in years (mean ± SD)	47.97 ± 9.72	46.7 ± 11.2	NS^**t**^
Gender			
Male, *n* (%)	19 (63.3)	26 (43.4)	NS^**C**^
Female, *n* (%)	11 (33.5)	34 (56.6)	
DM, *n* (%)	13 (43.3)	29 (48.3)	NS^**C**^
Duration of DM in months, median (IQR) months	72 (12–132)	120 (6–240)	NS^**w**^
HTN, *n* (%)	18 (60)	53 (88.3)	0.002^C^
Duration of HTN in months, median (IQR) months	60 (12–144)	120 (12–216)	NS^**w**^
Laboratory data			
Hb (gm/dL)	11.76 ± 1.48	10.9 ± 1.66	0.01^t^
CRP	25(15–63)	25 (7–96)	NS^w^
Phosphorus	3.88 ± 0.83	6.28 ± 1.73	> 0.001^t^
HCO3 (mEq/L)	20.39 ± 3.68	18.9 ± 3.8	NS^t^
Albumin	3.68 ± 0.47	3.55 ± 0.49	NS^t^
Parathyroid hormone (P.T.H) (pg/ml)	130 (64–363)	469 (58–1750)	> 0.001^w^
Calcium (mg/dl)	8.74 ± 0.76	8.31 ± 0.72	0.01^t^
Uric acid (mg/dl)	4.65 ± 0.94	6.36 ± 1.14	> 0.001^t^
GFR (ml/min/1.73 m^2^)	40 (31–57)	18 (3–29)	> 0.001^w^
FGF (ng/ml)	0.109 (0.06–0.14)	0.152 (0.1–0.28)	> 0.001^w^
24-h urinary phosphorus (mg/day)	1780 (645–4060)	1778 (245–5010)	NS ^w^
Nephron index	17933.77 ± 6413.76	12743.5 ± 6558.5	> 0.001^t^
Radiological assessment			
FMD percentage	0.15 (0.02–0.27)	0.08 (0–0.35)	> 0.**0**01^w^

*t* sample t test, *c* chi square test, *w* Mann–Whitney *U*, *NS* non-significant

Nephron index was the only parameter positively correlated to FMD change in stage 3 CKD (*r* = 0.47, *P* = 0.006). However, in CKD patients with creatinine clearance below 30 ml/min/1.73 m^2^, FMD change was positively albeit weakly correlated to nephron index (*r* = 0.28, *P* = 0.02) and measured GFR. In the same group, FMD change was negatively correlated to duration of HTN, serum phosphorus, PTH, serum bicarbonate, and serum FGF 23 levels (Table 2, Fig. 1).

**Table 2:** Correlation of demographic, clinical, and laboratory characteristics with the percentage of FMD in the studied groups

Variable	Percentage FMD			
	3		4–5	
	*r*	*P*	*r*	*P*
Age	− 0.14^s^	NS	− 0.13^s^	NS
Gender	− 0.008^s^	NS	− 0.05^s^	NS
DM	− 0.13^s^	NS	− 0.16^s^	NS
Duration of DM	0.3^s^	NS	− 0.15^s^	NS
HTN	0.3^s^	NS	− 0.07^s^	NS
Duration of HTN	0.4^s^	NS	− **0.34**^**s**^	**0.01**
CRP	− 0.03^s^	NS	0.13^s^	NS
Serum phosphorus	0.18^s^	NS	− **0.53**^**s**^	** > 0.001**
HCO3 (mEq/l)	− 0.15^s^	NS	− **0.39**^**s**^	**0.002**
Parathyroid hormone (P.T.H) (pg/ml)	0.001^s^	NS	− **0.53**^**s**^	** > 0.001**
Calcium (mg/dl)	0.17^s^	NS	0.13^s^	NS
Uric acid	− 0.21^s^	NS	− **0.5**^**s**^	** > 0.001**
GFR	0.21^s^	NS	**0.59**^**s**^	** > 0.001**
Serum FGF	− 0.31^s^	NS	− **0.38**^**s**^	**0.002**
24-h urinary phosphorus	− 0.31^s^	NS	0.12^s^	NS
Nephron index	**0.479**^**s**^	**0.006**	**0.284**^**s**^	**0.02**

Bold indicates the significant correlations

^s^Spearman correlation

In a multivariate linear regression analysis for predictors of FMD impairment in CKD stage 3 patients, the highest correlates were included as independent variables (serum phosphorus, iPTH, serum uric acid, measured GFR, nephron index). Of them, the nephron index was the only statistically significant predictor in the supposed regression model. In the patients with measured GFR below 30 ml/min/1.73 m^2^**,** none of the independent variables were related to the dependent outcome (Table 3).

**Table 3:** Multivariable linear regression model for the prediction of FMD percentage in the studied groups

Independent variable	Standardized coefficient (Beta)	*t*	Significance
GFR more than 30			
Constant		− 0.001	0.999
Serum phosphorus	− 0.117	− 0.549	0.588
iPTH	0.07	0.343	0.735
Measured GFR	0.219	1.167	0.254
Uric acid	0.064	0.327	0.747
Nephron index	**0.499**	**2.650**	**0.01**
GFR less than 30			
Constant		1.408	0.165
Serum phosphorus	− 0.156	− 0.748	0.458
iPTH	− 0.232	− 1.35	0.183
Measured GFR	0.107	0.404	0.688
Uric acid	− 0.07	− 0.405	0.687
Nephron index	0.072	0.474	0.637

Bold indicates the significant correlations

Dependent variable: FMD percentage

## Discussion

There is day-by-day growing evidence that CKD per se is one of the most important risk factors for cardiovascular morbidity and mortality [17]. Endothelial dysfunction is the primary step for the development of atherosclerosis [18]. In a previous report, the hazard ratio of developing ED in stage 3a CKD was reported to be 1.4 versus 3.4 in GFR below 15 [19]. ED in CKD can be contributed to complex pathophysiologic changes like chronic inflammation, mineral-bone disorders (MBD), anemia, oxidative stress, hyperuricemia, and uremic toxemia [20]. Many methods for ED assessment are available; the percentage of FMD relies on the direct relationship between the post-occlusion arterial dilation and integrity of vascular endothelium [21]. It was previously reported that the brachial FMD is reflective of ED in coronaries [22]. In another study, it was found to be correlated with CKD class [23], a finding which was not applied to our cohort except in stage 4–5 patients. This can be explained by the difference in population included, as we are just including stage 3 patients and the majority are stage 3b, not all the 5 CKD classes. Early prediction and management of ED are crucial in maintaining cardiovascular health. Many biomarkers were studied as predictors of ED in CKD like NO, eNOS, endothelin, endocan, and endothelial microparticles [21]. The latter is the most reliable marker of endothelial damage and is correlated to the impairment of FMD percentages in ESRD [24]. To our knowledge, there are no reliable markers of ED in earlier stages of CKD. Since they started to change very early in the course of CKD and progress with the GFR deterioration, markers of BMD were at the scope of cardiovascular and ED risk stratification research. Of them, FGF 23 gained the highest attention as its rise is known to precede the dominance of hyperphosphatemia and hyperparathyroidism [25]. FGF 23 was confirmed to be a major contributor to left ventricular hypertrophy through a klotho-independent pathway [6], but its relation to atherosclerosis, endothelial dysfunction, and vascular calcifications is questionable, especially at the early stages of CKD. In a survey of 183 CKD 3–4 patients, high FGF-23 is associated with vascular dysfunction and lower FMD. The authors suggested that this effect was referred to its possible endogenous inhibitory action on NO synthase [26]. In another cohort of 119 Japanese hemodialysis patients, FGF23 concentration was not associated with parameters of cardiac dysfunction, atherosclerosis, infection, and systemic inflammation [10]. In the current study, the FGF 23 levels were elevated in stage 3 CKD patients but not correlated to FMD percentage change in both univariate correlation and multivariate linear regression analysis. It was found to be correlated to the functional ED, as measured by FMD, in stage 4–5 CKD patients. Based on the phosphate-centric paradigm for the pathophysiology of CKD-related cardiovascular morbidity, urinary phosphorus excretion is supposed to augment the diagnostic utility of serum FGF 23 testing [11]. In a study including 880 patients with stable cardiovascular disease and normal kidney function to moderate CKD, greater urinary phosphorus excretion was associated with lower cardiovascular events and mortality [27]. In another cohort, urinary phosphate excretion was not correlated with the arterial stiffness index in CKD patients [28]. In the current study, we did not find a statistically significant correlation between 24-h urinary phosphorus excretion and indicator of ED. This can be logical in the context of the fact that phosphate regulation is determined by a dynamic balance between multiple factors including intake, absorption, and excretion. Even the excretion itself is based on the functioning nephron mass and the phosphaturic mediators. Thus, it was justified to combine the urinary phosphorus excretion to serum FGF 23 in an index to improve the diagnostic and prediction utility of both as it represents the sensitivity of the kidney to FGF23. During an average 7.5 years of follow-up of 872 outpatients with stable CVD and a mean estimated GFR of 71 ml/min per 1.73 m^2^, associations of FGF23 with mortality and cardiovascular events are stronger in persons with lower urinary phosphorus excretion independent of PTH and kidney function. In such individuals, the renal tubular response to FGF23 may be suboptimal [29]. In 142 diabetic-early CKD patients, a decrease in nephron index reflects early-stage renal impairment and is an independent risk factor of atherosclerosis in diabetic patients [13]. In another Indian cohort of 110 predialysis CKD, the nephron index was found to be correlated to atherosclerosis measured by carotid intimal media thickness [30]. To our knowledge, this study is the first to report a correlation between the nephron index and estimates of ED in CKD stage 3 patients. A relation that is attenuated at the later stages of CKD because of evident hyperphosphatemia, hyperparathyroidism, and hyperuricemia. The study has some limitations, firstly, its cross-sectional design that cannot establish a causation relationship; secondly, the non-inclusion of stage 2 CKD patients as it is known that the FGF 23 surge starts from stage 2 CKD.

## Conclusion

The Nephron index can be a more accessible tool for the prediction of ED in early CKD patients than the known complex biomarkers of ED which are not freely available. This index is a good estimate for the resistance of phosphaturic action of FGF 23 which is the primary step in the CKD-related cardiovascular effect.

## Data Availability

The datasets generated and/or analysed during the current study are available from the corresponding author on reasonable request.
